# Biocompatible Colloidal Suspensions Based on Magnetic Iron Oxide Nanoparticles: Synthesis, Characterization and Toxicological Profile

**DOI:** 10.3389/fphar.2017.00154

**Published:** 2017-03-28

**Authors:** Dorina-Elena Coricovac, Elena-Alina Moacă, Iulia Pinzaru, Cosmin Cîtu, Codruta Soica, Ciprian-Valentin Mihali, Cornelia Păcurariu, Victor A. Tutelyan, Aristidis Tsatsakis, Cristina-Adriana Dehelean

**Affiliations:** ^1^Faculty of Pharmacy, “Victor Babecs” University of Medicine and PharmacyTimişoara, Romania; ^2^Faculty of Medicine, “Victor Babeş” University of Medicine and PharmacyTimişoara, Romania; ^3^“George Emil Palade” Electron Microscopy Center, Institute of Life Sciences, “Vasile Goldiş” Western University of AradArad, Romania; ^4^Faculty of Industrial Chemistry and Environmental Engineering, Politehnica University of TimişoaraTimişoara, Romania; ^5^Federal Research Centre of Nutrition, Biotechnology and Food SafetyMoscow, Russia; ^6^Department of Forensic Sciences and Toxicology, Faculty of Medicine, University of CreteCrete, Greece

**Keywords:** bioavailability, skin barrier, toxicity, magnetic iron oxide nanoparticles, colloidal suspensions, solution combustion synthesis

## Abstract

The use of magnetic iron oxide nanoparticles in biomedicine has evolved intensely in the recent years due to the multiple applications of these nanomaterials, mainly in domains like cancer. The aim of the present study was: (i) to develop biocompatible colloidal suspensions based on magnetic iron oxide nanoparticles as future theranostic tools for skin pathology and (ii) to test their effects *in vitro* on human keratinocytes (HaCat cells) and *in vivo* by employing an animal model of acute dermal toxicity. Biocompatible colloidal suspensions were obtained by coating the magnetic iron oxide nanoparticles resulted during the solution combustion synthesis with a double layer of oleic acid, as innovative procedure in increasing bioavailability. The colloidal suspensions were characterized in terms of dynamic light scattering (DLS) and transmission electron microscopy (TEM). The *in vitro* effects of these suspensions were tested by means of Alamar blue assay and the noxious effects at skin level were measured using non-invasive methods. The *in vitro* results indicated a lack of toxicity on normal human cells induced by the iron oxide nanoparticles colloidal suspensions after an exposure of 24 h to different concentrations (5, 10, and 25 μg·mL^−1^). The dermal acute toxicity test showed that the topical applications of the colloidal suspensions on female and male SKH-1 hairless mice were not associated with significant changes in the quality of barrier skin function.

**Figure:**
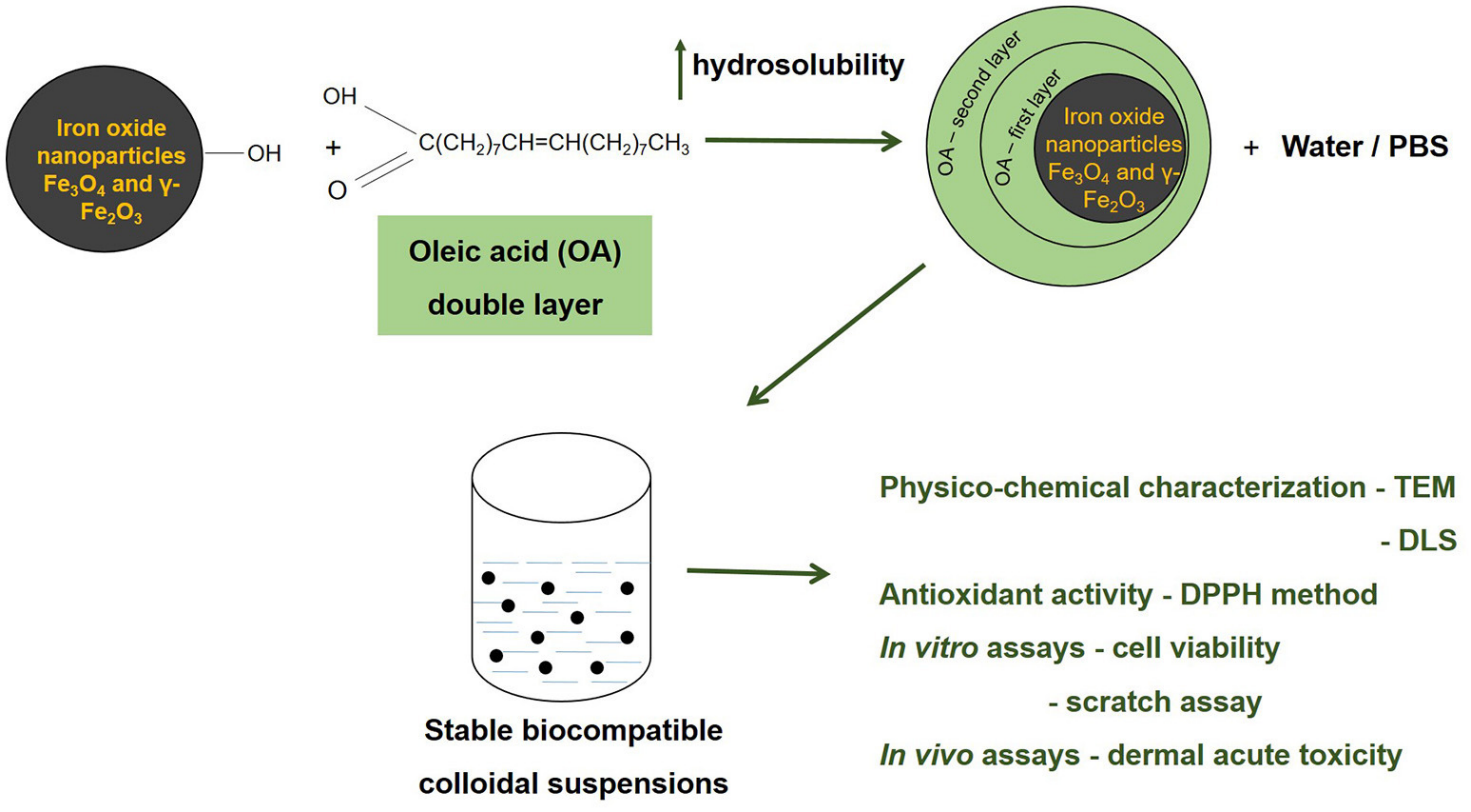
GRAPHICAL ABSTRACT

## Introduction

The field of nanotechnology has known a considerable progress in recent years, mainly regarding its involvement in different biomedicine applications. There were recorded extensive efforts to develop effective nanomaterials as diagnostic, prevention and treatment tools for cancer and other disorders, such as infectious, cardiovascular, and central nervous system diseases (Zdrojewicz et al., 2015; Radomska et al., 2016; Torres-Sangiao et al., 2016). The interest for the liaison between nanoparticles and medicine could be quantified by the number of articles enlisted in the PubMed database—over 130,000 at the moment that this article was written (November 2016), number acquired after a search for “nanoparticles.”

From the great number of nanoparticle types, magnetic nanoparticles attracted an increased attention due to their potential use in multiple medicine branches, such as: magnetic resonance imaging, thermal therapy, cell labeling, drug, and gene targeted delivery, *in vitro* diagnosis, immunoassays, nucleic acid concentration (reviewed in Gobbo et al., 2015; Medeiros et al., 2015). The group of magnetic nanoparticles includes: pure metals (iron, nickel, and cobalt), metal alloys or metal oxides (Conde et al., 2014; Soares et al., 2016). Iron oxide nanoparticles are the highest ranked nanomaterials in medicine due to their unique physico-chemical properties (superparamagnetism) and their established biocompatibility and stability in aqueous solutions (Medeiros et al., 2015; Soares et al., 2016, reviewed in Valdiglesias et al., 2016). Iron oxide nanoparticles were employed for multiple clinical applications, including: magnetic resonance imaging (MRI) as contrast agents, drug carrier platforms for anticancer agents, magnetic cell separation, in high-gradient magnetic field separations, treatment of retinal detachment, bio-catalysis, and protein purification (Shete et al., 2015; Tran et al., 2015). Besides this, bioavailability of orally introduced Fe (III) oxide nanoparticles (in form of hematite) was characterized in male Wistar rats (Raspopov et al., 2011). Nanoparticles of ferric oxide with mean size 13.4 nm were capable to restore iron deposits of animals impaired as a result of iron-deficient diet consumption.

A plus of the iron oxide nanoparticles consists in the fact that this type of nanoparticles are not charged or aggregated at physiological pH due to their isoelectric point—7; aggregated nanoparticles being easily detected by the immune cells and cleared from the organism before they could fulfill their assignment (Tran et al., 2015).

For biomedical uses are considered adequate only the magnetic nanoparticles that comply with the following requirements: superparamagnetic properties at room temperature, a wide saturation magnetization, a size in the range of 20 nm for *in vivo* administration and to be biocompatible (Shete et al., 2015). In order to convert the pure magnetic nanoparticles into biocompatible dispersions it was proposed the use of different polymers as capping agents or surfactants, like: starch, chitosan, dextran, oleic acid, polyethylene glycol, etc. (Józefczak et al., 2012; Medeiros et al., 2015; Shete et al., 2015).

For the synthesis of iron magnetic nanoparticles was proposed an array of methods, including: mechanical grinding, arc discharge, high temperature decomposition of organic precursors, chemical co-precipitation, laser ablation, gas deposition, electron beam lithography, reverse micelles method, hydrothermal method, microemulsion, solvothermal method, solution combustion synthesis, etc. (reviewed in Gupta and Gupta, 2005; Sun et al., 2007; Li et al., 2010; Ianoş et al., 2012; Velusamy et al., 2016). Among all these methods, solution combustion synthesis was proved to be a promising alternative for the preparation of a considerable number of metal oxide nanopowders with multiple benefits: short preparation time, low energy consumption, cheap starting materials, self-sustained reaction, high effectiveness, simple procedure and low cost apparatuses and suitability for mass production (Ianoş et al., 2012; Huang et al., 2016).

In the present study, biocompatible colloidal suspensions of magnetic iron oxide nanoparticles coated with oleic acid were prepared and characterized. There were investigated the effects of these nanoparticles both *in vitro* on normal cell lines —human keratinocytes (HaCat cells) and *in vivo* by evaluating the acute dermal toxicity after topical application of the colloidal suspensions.

## Materials and methods

### Materials

#### Chemicals and reagents

The reagents used for the synthesis of Fe_3_O_4_ (magnetite) and γ-Fe_2_O_3_ (maghemite) nanoparticles and of the colloidal suspensions were: Fe(NO_3_)_3_·9H_2_O (Roth)—as oxidizing agent, C_6_H_8_O_7_·H_2_O (Silal Trading) and D-(+)-C_6_H_12_O_6_ (Riedel de Haën)—as fuels and oleic acid—C_18_H_34_O_2_ (Merck 65–88%)—as surfactant. The cell culture media—Dulbecco's modified Eagle Medium with high glucose and the other chemicals used for cell culture: Fetal Bovine Serum (FBS), penicillin/streptomycin, PBS (phosphate saline buffer), Trypsin/EDTA, Trypan Blue, and Alamar blue solutions were bought from Sigma Aldrich, Germany. The ethanol was purchased from Chimreactiv-Bucharest, Romania. All chemicals were of the highest grade of purity commercially available.

#### Cells

The cell line used in this study was of human origin: human keratinocytes—HaCat cells (was offered by the University of Debrecen, Hungary). The cell line was kept in standard conditions before culture (liquid nitrogen).

#### Animals

The animals used for the acute dermal toxicity test were female and male SKH-1 hairless mice purchased from Charles River Laboratories (Budapest, Hungary) and kept in the university animal facility.

### Methods

#### Synthesis of Fe_3_O_4_ nanoparticles

The magnetite and maghemite nanoparticles used for the preparation of colloidal suspensions were synthesized using a new version of the solution combustion synthesis (Ianoş et al., 2012, 2014). The aqueous solution containing Fe(NO_3_)_3_·9H_2_O and C_6_H_8_O_7_·H_2_O (fuel for the synthesis of magnetite), D-(+)-C_6_H_12_O_6_ (fuel for the synthesis of maghemite) respectively, was heated to 400°C in the absence of air, in a round bottom flask. As the water evaporated, a smoldering combustion reaction occurred, leading to the formation of a black powder. The resulted black powder was hand crushed, washed with warm distilled water and dried at 80°C (for samples S1 and S2). The black powder resulted by using glucose as fuel was further treated with H_2_O_2_ in order to remove the residual carbon present on the surface of particles after combustion reaction (for sample S3).

#### Preparation of stable colloidal suspensions

The protocol used for the preparation of the colloidal suspensions by coating with a double layer of oleic acid, was a modified version of the protocol described by Bica et al. (2007). In brief, the iron oxide nanoparticles prepared by solution combustion synthesis were sonicated for several hours and then covered with a double layer of oleic acid. For the coating process it was used an excess of oleic acid (2:1 ratio). The residual oleic acid and the other salts were eliminated by decantation and it was obtained a dispersion of oleic acid double layer coated iron oxide nanoparticles, that was further dissolved in phosphate buffered saline (PBS—sample S1) and in distilled water (samples S2 and S3) respectively, leading to stable colloidal suspensions.

#### Characterization of the iron oxide nanoparticles

Iron oxide nanoparticles were characterized in terms of XRD phase composition (Rigaku Ultima IV, Cu_Kα_, Tokyo, Japan), specific surface area (nitrogen adsorption-desorption, Micromeritics ASAP 2020, Micromeritics Instrument Corporation, Norcross, USA) and magnetic properties (VSM 880 ADE/DMS magnetometer DMS/ADE Technologies, Massachusetts, USA).

#### Characterization of the colloidal suspensions

The resulted colloidal suspensions solubilized in PBS (sample S1) and in distilled water (samples S2 and S3) were characterized by dynamic light scattering—DLS, using a ZetaSizer NanoZS Malvern Instrument (Worcestershire, UK). The morphology and ultrastructure of aggregates and nanoparticles were characterized by transmission electron microscopy (TEM) using a FEI Tecnai 12 Biotwin, (Oregon, SUA) electron microscope. The samples obtained as described in the previous section were sonicated for 15 min. After the sonication, 1 drop from each sample was placed on the copper grid surface. This method was used to determine the size of the nanoparticles.

#### Antioxidant activity of the iron oxide colloidal suspensions

The antioxidant activity of the samples was evaluated by DPPH radical scavenging assay which was originally described by Blois (1985). The solution of DPPH (1 mmol·L^−1^) was used as a standard reagent. As control, it was prepared a solution of ascorbic acid (from Lach-Ner; 0.167 mmol·L^−1^) in ethanol 96%. From each sample (S1, S2, S3) there were made three dilutions: 1:10; 1:50; 1:100, as presented in Table 1.

**Table 1:** **Samples S1, S2, and S3 concentrations after dilution**.

**Sample**	**Initial concentration, C_*i*_ (mg·mL^−1^)**	**Dilution**	**Final concentration, C_*f*_ (mg·mL^−1^)**
S1	10.4	1:10	1.04
		1:50	0.208
		1:100	0.104
S2	52	1:10	5.2
		1:50	1.04
		1:100	0.52
S3	78	1:10	7.8
		1:50	1.56
		1:100	0.78

A mixture of: 0.5 mL of each dilution, 2 mL ethanol 96% and 0.5 mL DPPH ethanolic solution was analyzed using a T80UV/VIS Spectrophotometer (PG Instruments LtD) at 516 nm for 20 min.

Antioxidant activity was calculated using the following formula:

$$\begin{array}{l} AOA\left(\%\right)=100-\frac{A_{t_{516}}}{A_{{\left(t=0\right)}_{516}}}\cdot 100 \end{array}$$

Where: AOA = antioxidant activity

$$\begin{array}{ll} A_{t_{516}} & =\text{absorbance of the sample }\left(\text{S}1,\text{ S}2,\text{ S}3\right)\text{ measured at}\\ \;516\text{ nm at a specific time}\\ A_{{\left(t=0\right)}_{516}} & =\text{absorbance of the blank solution measured at}\\ \;516\text{nm }\left(\text{without test sample}-\text{S}1,\text{ S}2,\text{ S}3\right). \end{array}$$

#### Cell culture

The human keratinocytes—HaCat were cultured in Dulbecco's modified Eagle Medium (DMEM) with high glucose (4.5 g·L^−1^), 15 mM Hepes, and 2 mM L-glutamine, supplemented with 100 U·mL^−1^ penicillin, 100 μg·mL^−1^ streptomycin, and 10% fetal bovine serum (FBS). The cells were kept in standard conditions: a humidified atmosphere with 5% CO_2_ at 37°C and were passaged every 2 days. Cell number was determined using the cell counting chamber—Neubauer in the presence of Trypan blue.

#### Viability assay—alamar blue

The viability was measured using the Alamar blue technique, a classical assay to measure the cytotoxicity induced by different agents. The cells (1 x 10^4^/200 μL medium/well) were seeded in a 96-well plate and allowed to attach. After the cells attached to the plate, were stimulated with different concentrations (5, 10, and 25 μg·mL^−1^) of the 3 colloidal suspensions for 24 h. Before the Alamar blue reagent be added, the medium that contained the suspensions was removed and new medium was added into each well—200 μL/well (the iron nanoparticles could interfere with the spectrophometrical measurement of Alamar blue and the results might have been false positive). At 24 h post-stimulation, it was added a volume of 20 μL/well of Alamar blue solution (10% of the volume of cell culture medium present in each well—200 μL). The plates were incubated for 3 h at 37°C, followed by the measurement of the absorbance using xMark™Microplate Spectrophotometer (Biorad). Cell viability was calculated according to the formula used in one of our previous articles (Şoica et al., 2014).

#### Scratch assay

This assay represents an *in vitro* wound healing assay and was used to determine how fast a generated gap in the monolayer can be repopulated by migrating cells, and to define differences in migration or proliferation under various experimental conditions.

The scratch assay protocol was applied according to the data from the literature (Jung et al., 2012): 2 × 10^5^ cells/well were seeded in 12-well culture plates 1 day prior to the experiment. Scratches were performed in a defined area using a small pipette tip (10 μL). Detached cells and medium were removed by washing with PBS prior to treatment. Cells were observed over a period of 24 h. Photos of the scratches were taken at different time points (0, 3, and 24 h) using an Optika Microscopes Optikam Pro Cool 5 and Optika View.

#### *In vivo* acute dermal toxicity

The experimental procedures and protocols that were applied in the present study were in agreement with the European Directive 2010/63/EU and the National Law 43/2014 regarding the protection of animals used for scientific purposes. The experimental protocol was approved by the Committee for Research Ethics of “Victor Babeş” University of Medicine and Pharmacy, Timişoara, Romania. The animals received food and water *ad libitum* and were kept in the University animal facility under standard conditions [constant temperature of 22.5 ± 2°C and relative humidity of 55% ± 5%, 12 h (light)—12 h (dark) cycle]. The animals used in the study were adult female and male SKH-1 hairless mice (*n* = 5/group). The acute dermal toxicity was tested according to the OECD guideline 402. The female mice were non-pregnant. Before the experiment, the mice were acclimatized to the laboratory conditions for 2 weeks and were divided in 3 groups/sex: group 1—mice that received topically the colloidal suspension of magnetite—S1 (in PBS), group 2—mice that received topically the colloidal suspension of magnetite—S2 (in distilled water) and group 3—mice that received topically the colloidal suspension of maghemite—S3 (in distilled water). The volume of colloidal suspension topically administered was 100 μL/3 times at an interval of 20 min between applications. These volumes were applied on the first day of experiment and the animals were monitored for a period of 14 days. The parameters evaluated were: changes in mice weights, behavioral pattern (salivation, tremors, lethargy, sleep, and coma) and changes in skin appearance by measuring the physiological skin parameters values.

#### Non-invasive skin parameters measurements

The skin parameters were measured by the means of a non-invasive technique using the equipment from Courage-Khazaka, Germany: an electronic Skin Colorimeter CL 400 and the Corneometer®CM 825. The Skin Colorimeter CL 400 principle is based on the tristimulus colorimetry and uses the Commission Internationale de l'Eclairage (CIE) L^*^a^*^b^*^ color system to determine skin color modifications. The color was expressed using the parameters L^*^a^*^b^*^, where: L^*^ measured skin reflectance or lightness (a gray scale with values ranging from 0 to 100 where 0 is black and 100 is white); a^*^ measures the colour saturation from red to green (scale from +60 to −60, where positive values indicate varying intensities of red); b^*^ measures the color saturation from yellow to blue (scale +60 to −60, where positive values indicate varying intensities of yellow; Alaluf et al., 2002). The L^*^a^*^b^*^ parameters were expressed as arbitrary units. The hydration of the stratum corneum was determined using the Corneometer®CM 825 probe.

#### Statistical analysis

The statistical programs and softwares applied in the present study were GraphPad Prism 5 and Origin 8 (OriginLab—Data analysis and Graphing Software).

## Results

### Characterization of the colloidal suspensions based on iron oxide nanoparticles

The colloidal suspensions (magnetite—samples S1 and S2 and maghemite—sample S3) doubled coated with oleic acid were prepared using the iron oxide nanoparticles obtained during the solution combustion synthesis. The coating process occurred after the solution combustion synthesis using oleic acid in excess as compared to the amount of nanopowder (2:1 ratio).

The main characteristics of the samples used in the present study were presented in Table 2.

**Table 2:** **Characteristics of the colloidal suspensions based on iron oxide nanoparticles**.

**Powder no**.	**The combustible used in combustion reaction**	**The phase composition obtained**	**The density of the colloidal suspension (g/cm^3^)**	**The density of the dispersion medium (g/cm^3^)**	**The mass of iron oxide calculated / ml suspension (g)**
S1	C_6_H_8_O_7_·H_2_O Citric acid	Fe_3_O_4_	1.0126	PBS—1.0044	0.0104
S2	C_6_H_8_O_7_·H_2_O Citric acid	Fe_3_O_4_	0.9956	Water—0.9465	0.0520
S3	C_6_H_12_O_6_ Glucose	γ-Fe_2_O_3_	1.0097	Water—0.9465	0.078

The density of the colloidal suspension and the density of dispersion medium (distilled water or PBS) were measured using a Portable Density Meter: DMA 35 from Anton Parr at 25°C.

The volume fraction was calculated with the following formula:

$$\begin{array}{l} \varphi =\frac{\rho_{CS}-\rho_{\text{DM}}}{5.2-\rho_{\text{DM}}} \end{array}$$

Where:  ρ_CS_- the density of coloidal suspension
        ρ_DM_ - the density of dispersion medium
        5.2- the density of F *e*_3_*O*_4_

The mass of iron oxide was calculated by multiplication of the volume fraction of each colloidal suspension with 5.2 and with 1 mL of each colloidal suspension used.

**Figure 1:**
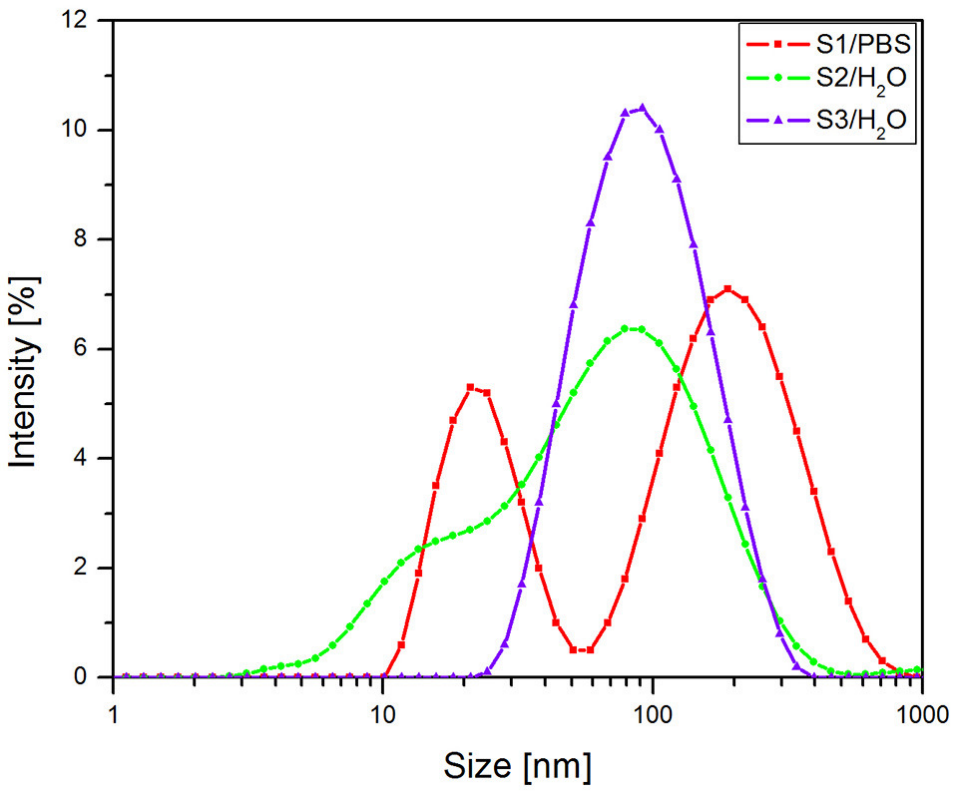
**The intensity distribution of particles size (DLS) of colloidal suspensions**.

In order to assess the particles size and distribution it was applied the dynamic laser scattering (DLS) method. The results recorded for the samples S2 and S3 (Figure 1) in terms of intensity distribution of particles size revealed that the colloidal suspensions were composed virtually of a single family of particles with an average hydrodynamic diameter of 43 nm (S2—green line) and 80 nm (S3—blue line), respectively. On the other hand, the sample S1 (red line Figure 1), presented a bimodal particle size distribution, which suggests the presence of two populations of particles with an average hydrodynamic diameter of 63 nm.

**Figure 2:**
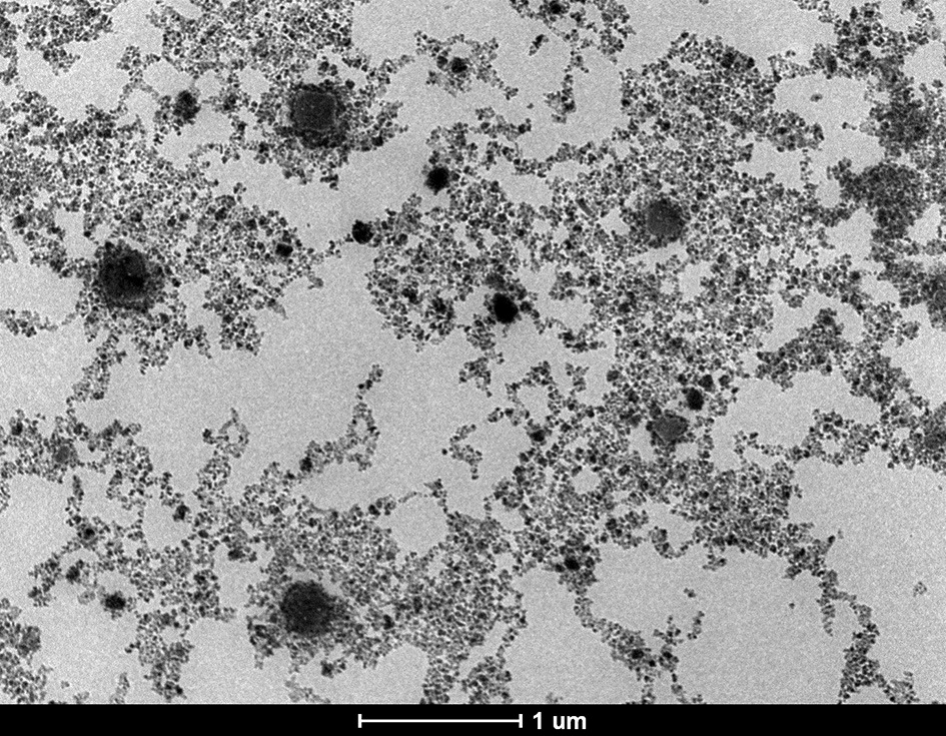
**TEM micrograph of the sample S2, general aspect of aggregates in rather low magnification (16,500x)**.

**Figure 3:**
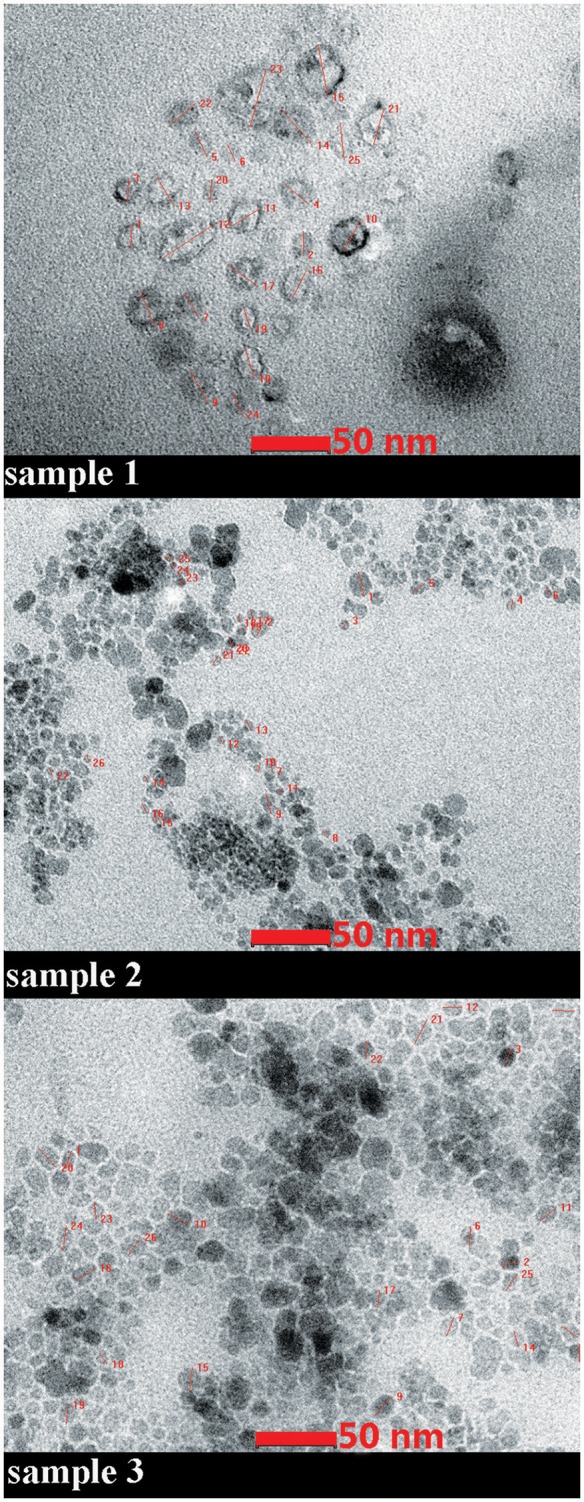
**TEM micrograph of the samples**. High magnification (220Kx). The details of measurements of nanoparticles size are given in red.

The data concerning the morphology and the ultrastructure of the colloidal suspensions were provided by the Transmission Electron Microscopy (TEM) analysis. The size and shape of nanoparticles were analyzed both in rather low magnification (16,500x) and in higher magnification (220Kx). The analysis of sample S2 in rather low magnification indicated uniformly distributed aggregates (Figure 2), whereas in higher magnification (220Kx) the nanoparticles were monodispersed with polygonal shapes and an average size between 7 and 22 nm (Figure 3).

TEM data recorded for sample 1 (magnetite double coated with oleic acid solubilized in PBS) in high magnetization showed a different shape of the nanoparticles and a larger size as compared to the ones measured in sample 2 (magnetite double coated with oleic acid solubilized in distilled water; see Figure 3). In terms of homogenous size and distribution of the nanoparticles, all three samples analyzed presented these characteristics (Figure 3).

**Figure 4:**
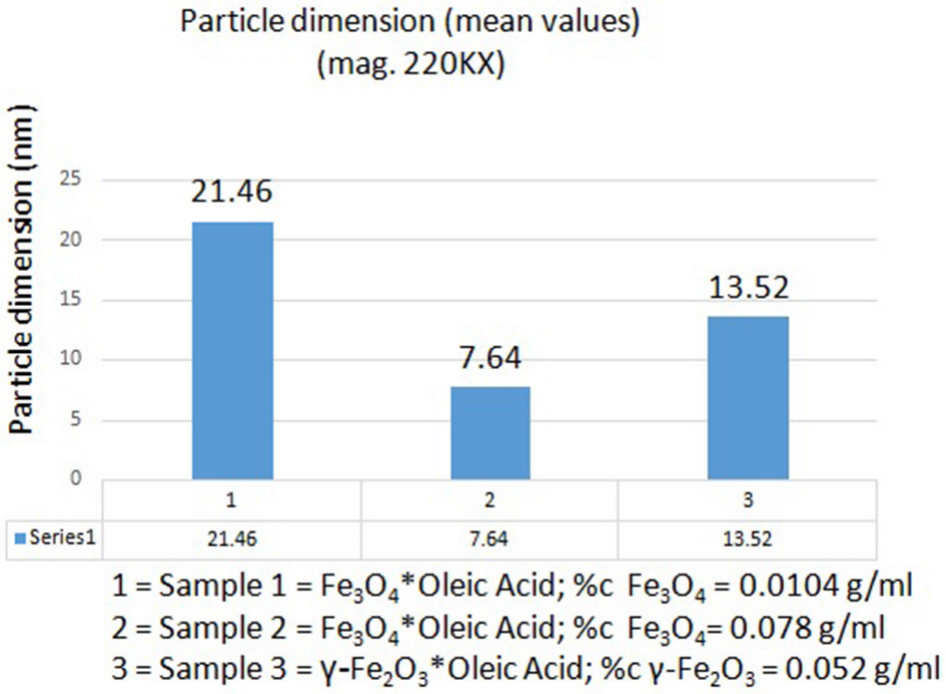
**The inverse correlation between the iron oxide concentration in samples and nanoparticle dimensions**.

According to the measurements (in high magnification) in all three samples (S1, S2, and S3), it could be observed an inverse correlation between the iron oxide concentration in the sample and the nanoparticle size. In the samples with a higher concentration of iron oxide it was observed a decreased value of nanoparticle size (7.64 nm–sample S2 and 13.52 nm–sample S3), whereas in the sample with a lower concentration in Fe_3_O_4_ (sample S1), the nanoparticles had an increased size (21.46 nm; see Figure 4).

### Antioxidant activity of the iron oxides colloidal suspensions

The antioxidant activity of the samples was evaluated by DPPH radical scavenging assay. There were prepared three dilutions for each sample (see Table 1) and tested for antioxidant activity.

**Figure 5:**
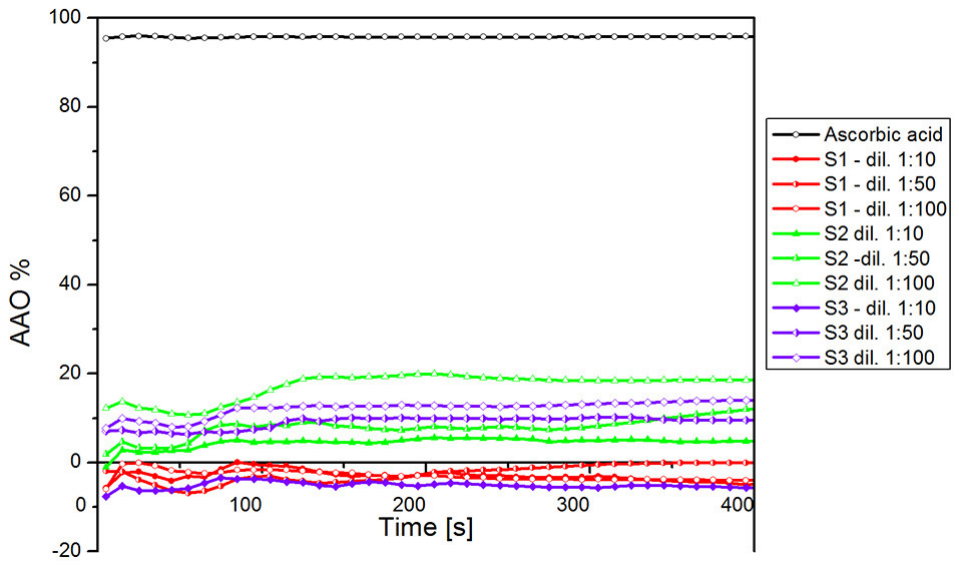
**AOA of all three samples of iron oxide colloidal suspensions, including the dilutions**.

In Figure 5 was presented the antioxidant activity (AOA) of all three samples of biocompatible magnetic colloidal suspension based on iron oxide nanoparticles. The antioxidant activity assessment was carried out for 1,200 s, but we chose to represent only the data recorded until 400 s during this time being detected most of the activity (after this time point no significant activity was detected until the end of experiment).

According to our results, all the samples showed a reduced antioxidant activity as compared to the AOA of ascorbic acid, used as positive control. Furthermore, sample S1 (magnetite—Fe_3_O_4_ nanoparticles dispersed in PBS) did not exhibit antioxidant activity for any dilution analyzed. In the case of sample S3 (maghemite—γ-Fe_2_O_3_ nanoparticles dispersed in water), the first dilution analyzed (1:10) did not reveal an antioxidant activity, but at higher dilution the AOA of the sample achieved a value of ~20%. This could be explained by the fact that this sample had the highest concentration in γ-Fe_2_O_3_ and a dilution of 1:10 could be too concentrated to present antioxidant activity.

The sample S2 (Fe_3_O_4_ nanoparticles dispersed in distilled water)—contained the same type of nanoparticles (Fe_3_O_4_) as sample S1, but the differences between S1 and S2 were the amount of Fe_3_O_4_ (S1—10.4 mg vs. S2—52 mg) and the liquid carrier (PBS/distilled water); this was the only sample that showed antioxidant activity for each dilution. The fact that the sample (S2) showed antioxidant activity compared with the sample S1 could be explained by the liquid carrier—any compound dispersed in water shows a higher antioxidant activity compared to the same compound dispersed in other liquid carrier, and by the amount of Fe_3_O_4_ in the sample.

**Figure 6:**
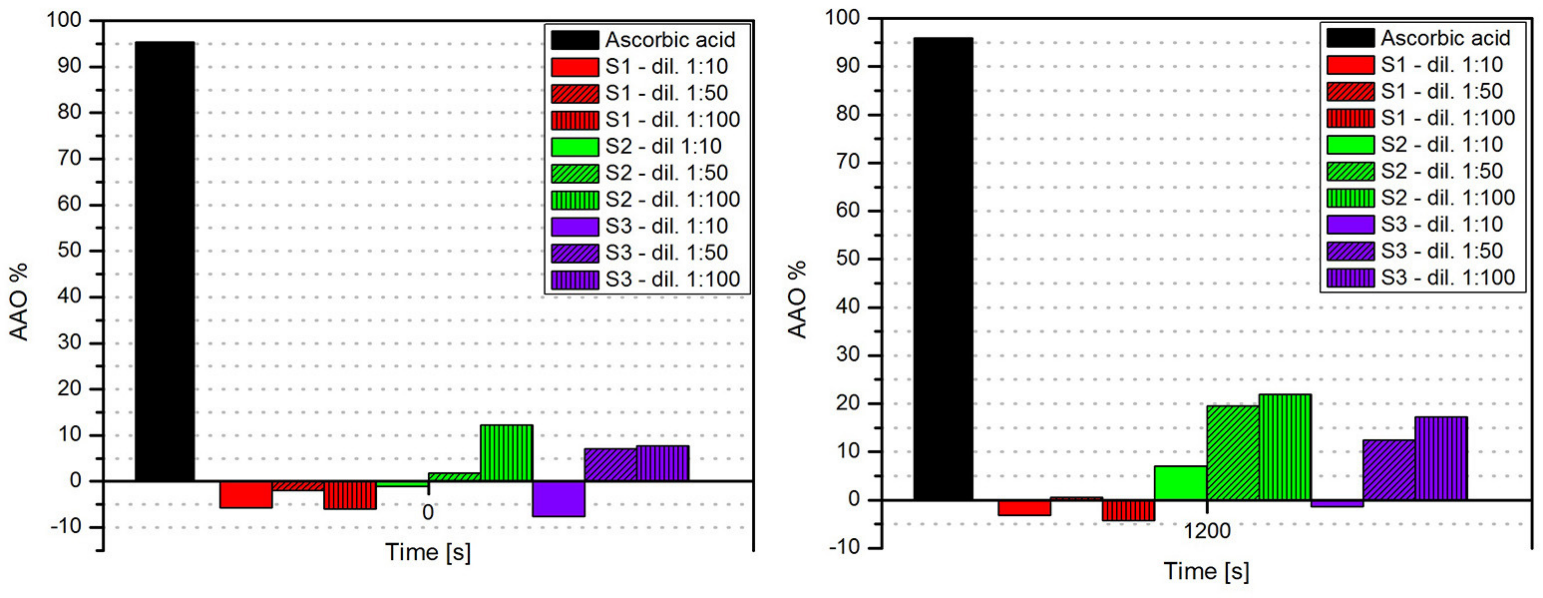
**The AOA of the samples at initial (*t* = 0 s) and at the final (*t* = 1,200 s) moment**.

In Figure 6 was presented the AOA of the samples both at initial and at the final moment. As it could be seen, the AOA (for each dilution) increased in time, this increase being in an inversely proportional relation with the samples concentration. This could be explained by the fact that a colloidal suspension based on iron oxides shows antioxidant activity only when the concentration in Fe_3_O_4_ or γ-Fe_2_O_3_ was higher and the solid fraction was dispersed in distilled water.

### Evaluation of magnetic colloidal suspensions effects on cell viability

Cells viability was expressed as percentage of viable cells (%) related to the control cells (the cells that were stimulated with the same concentration of solvent—for sample—S1—PBS and for samples—S2 and S3—distilled sterile water). This parameter was assessed by the means of Alamar blue technique.

Stimulation of the HaCat cells with different concentrations (5, 10, and 25 μg·mL^−1^) of samples S1, S2, and S3 colloidal suspensions for 24 h led to an increase of cell viability as compared to control cells, what indicated that the iron nanoparticles did not affect cells viability (Table 3). Similar results were obtained for a higher concentration (50 μg·mL^−1^) in the case of all three test samples—S1, S2, and S3 (data not shown).

**Table 3:** ***In vitro* effect of samples S1, S2, and S3 after 24 h stimulation**.

**Samples**	**Percentage of viable HaCat cells (%)**			
	**PBS/H_2_O**	**5 μg·mL^−1^**	**10 μg·mL^−1^**	**25 μg·mL^−1^**
S1	100	111.47	101.49	109.26
S2	100	118.45	129.15	102.78
S3	100	131.38	128.11	119.67

### Effects of magnetic colloidal suspensions on cell migration and proliferation

The effect of the magnetic iron oxide nanoparticles colloidal suspensions on cell migration and proliferation was assessed by the means of scratch assay, a wound healing type technique. After the scratches were drawn (when the confluence of the cells was around 90%), the cells were stimulated for 24 h with the same concentrations tested for the cytotoxic effect. There were taken pictures at different time points (0, 3, and 24 h) in order to pursue the impact of the test suspensions on cells migration and proliferation.

**Figure 7:**
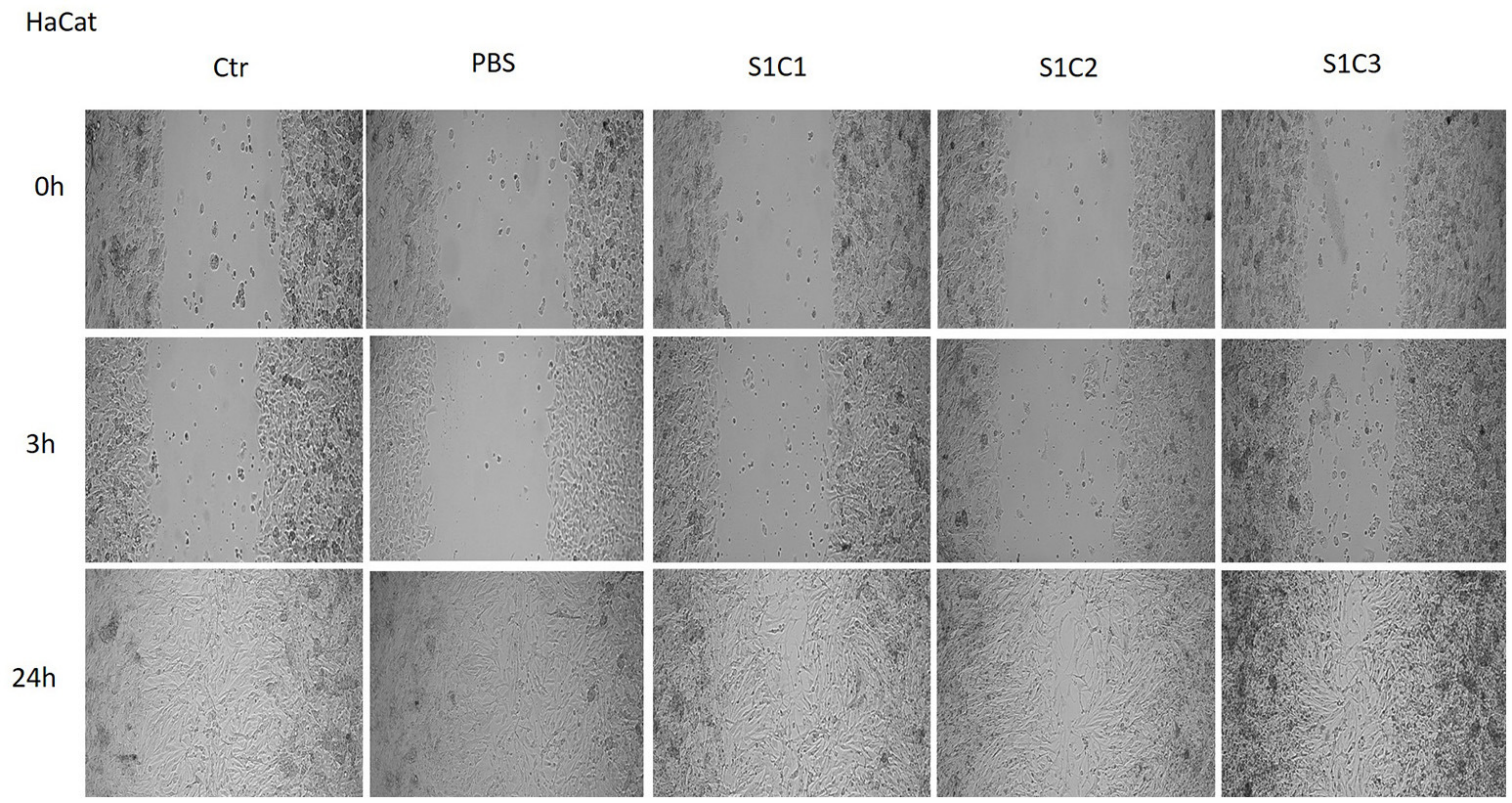
**The effect of S1 colloidal suspension on HaCat cells migration and proliferation**. The cells were stimulated with different concentrations of the colloidal suspension (C1 = 5, C2 = 10, and C3 = 25 μg·mL^−1^) and were taken photos at 0, 3, and 24 h post-stimulation.

As it can be seen in Figure 7, the S1 colloidal suspension did not affect the normal keratinocytes—HaCat migration, neither after 3 h, nor after 24 h, moreover, could it be said that the test suspension had a stimulatory effect on cells proliferation. The cells were abundant on the plate and well attached.

**Figure 8:**
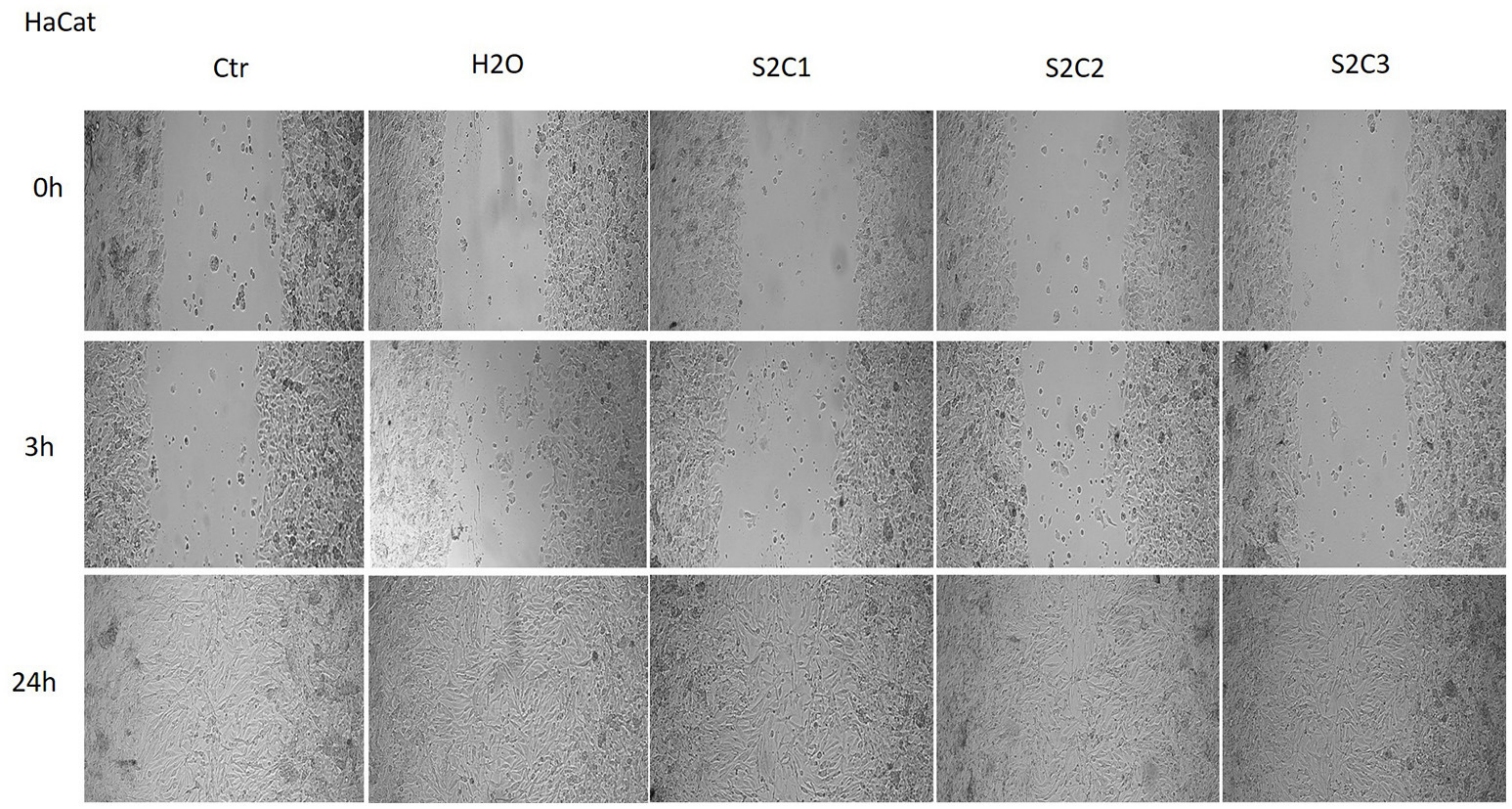
**The effect of S2 colloidal suspension on HaCat cells migration and proliferation**. The cells were stimulated with different concentrations of the colloidal suspension (C1 = 5, C2 = 10, and C3 = 25 μg·mL^−1^) and were taken photos at 0, 3, and 24 h post-stimulation.

**Figure 9:**
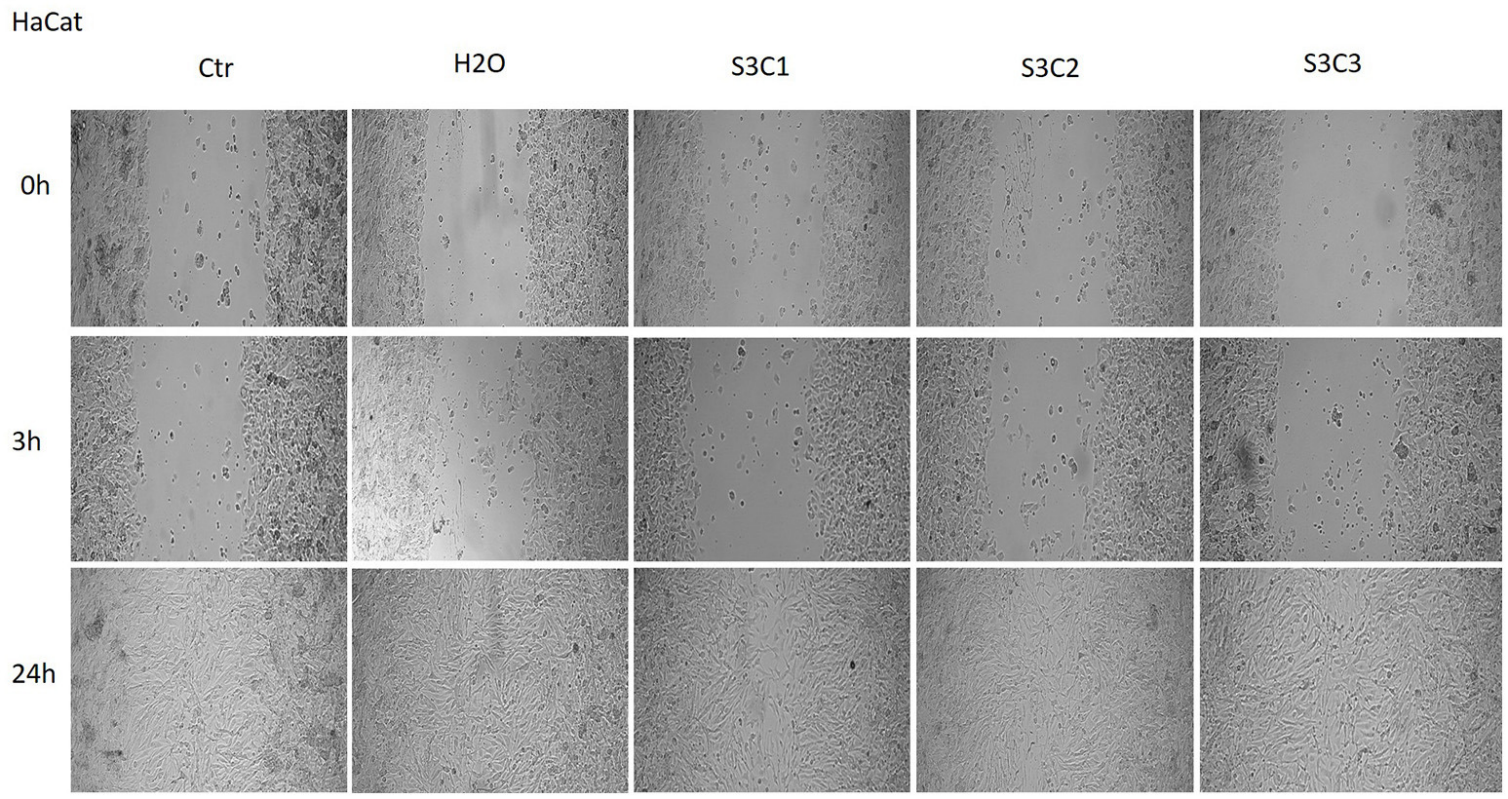
**The effect of S3 colloidal suspension on HaCat cells migration and proliferation**. The cells were stimulated with different concentrations of the colloidal suspension (C1 = 5, C2 = 10, and C3 = 25 μg·mL^−1^) and were taken photos at 0, 3, and 24 h post-stimulation.

The Figures 8, 9 showed that the samples S2 and S3 colloidal suspensions induced a similar effect on HaCat cells migration and proliferation as the one described for sample S1: a stimulatory effect, results that were in agreement with the data recorded for the cytotoxicity test.

### *In vivo* evaluation of acute dermal toxicity

The *in vivo* effects of the colloidal suspensions of magnetite and maghemite coated with a double layer of oleic acid were tested employing the OECD guideline 402 protocol for acute dermal toxicity. The mice used in the study were female and male SKH-1 hairless mice. The body weights of the mice were recorded every 2 days for 14 days and no significant modifications were observed neither in the female groups, nor in male groups. Concerning the behavioral patterns (salivation, lethargy, sleep, and coma), there were observed no such signs in any groups of mice during the experiment frametime. These data indicated that topical applications of iron colloidal suspensions were not associated with weight loss or interferences at nervous system level.

Another parameter that was assessed was the skin appearance after topical application of the iron colloidal suspensions. The changes in skin appearance were characterized by monitoring the evolution of skin physiological parameters—skin hydration, melanin content and erythema using a non-invasive technique. The measurements were performed before the application of the colloidal suspensions (values considered as controls), after each application (at 20, 40, and 60 min), at 6 h, 24 h and in every second day until the last day of experiment (day 14). The most significant changes were observed in the first 24 h. Until the end of experiment the values were constant (data not shown).

**Figure 10:**
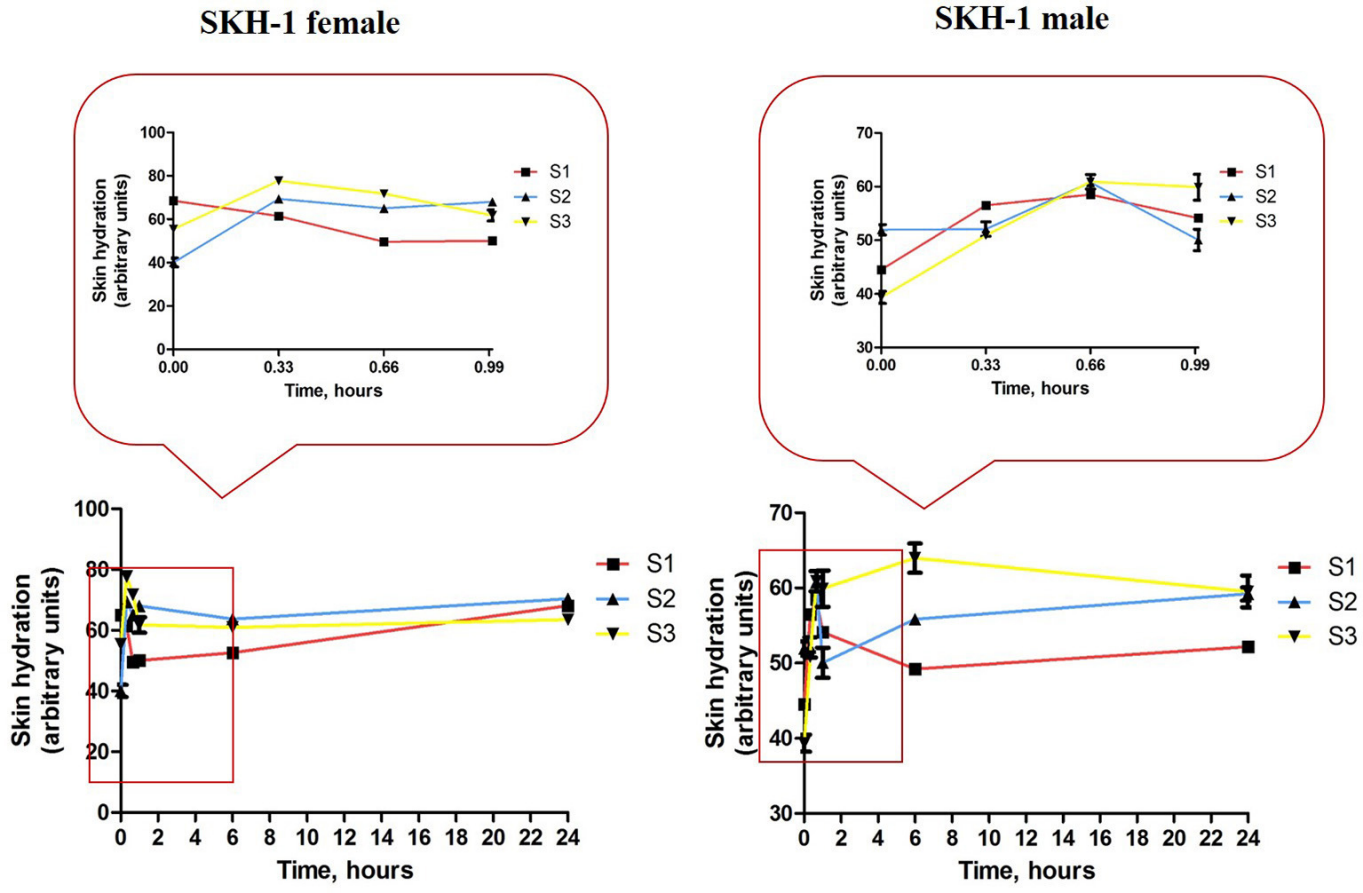
**Skin hydration evolution during the first 24 h of experiment**. Data are expressed as mean of the values measured at every time point (three successive measurements of the parameter/mouse).

Topical application of the iron colloidal suspensions led to some changes in the values of skin hydration both in the female and male groups of mice (Figure 10). S1 colloidal suspension in PBS (red line) led to a decrease in the skin hydration value in the females group with each application (in the first 1 h), but the measurements at 6 h and 24 h indicated a similar value to the one measured before the application of the colloidal suspension (control value) and this value continued in the same range until the end of experiment. In the case of S2 and S3 iron colloidal suspensions in distilled water (blue and yellow lines) it was detected an increase of the parameter in the first hour, this hydration status being maintained in the first 24 h.

Group 1 of male mice (S1 treated—red line) indicated an increase of skin hydration value in the first hour (after the 3 successive applications), but after 6 h the value of the parameter returned at the initial value (before topical application) and continued in this range until the end of experiment. When were applied S2 and S3 iron colloidal suspensions the evolution of skin hydration in the groups of male mice was similar with the one observed in the female groups—an increase value in the first hour and this value was maintained in the first 24 h.

Melanin content and the erythema were measured by the means of tristimulus colorimetry using the skin colorimeter CL 400 probe (Courage–Khazaka, Germany).

The iron colloidal suspensions were applied in the posterior thorax region of the mice (females and males) and the tristimulus colorimetric L^*^a^*^b^*^ measurements were recorded before each application (in the first hour), at 6 h, 24 h, and every 2 days until the end of the experiment. There were analyzed the values for L^*^–skin reflectance (lightness) and b^*^–yellowness, associated with melanin content, and a^*^–redness, an indicator for erythema.

**Figure 11:**
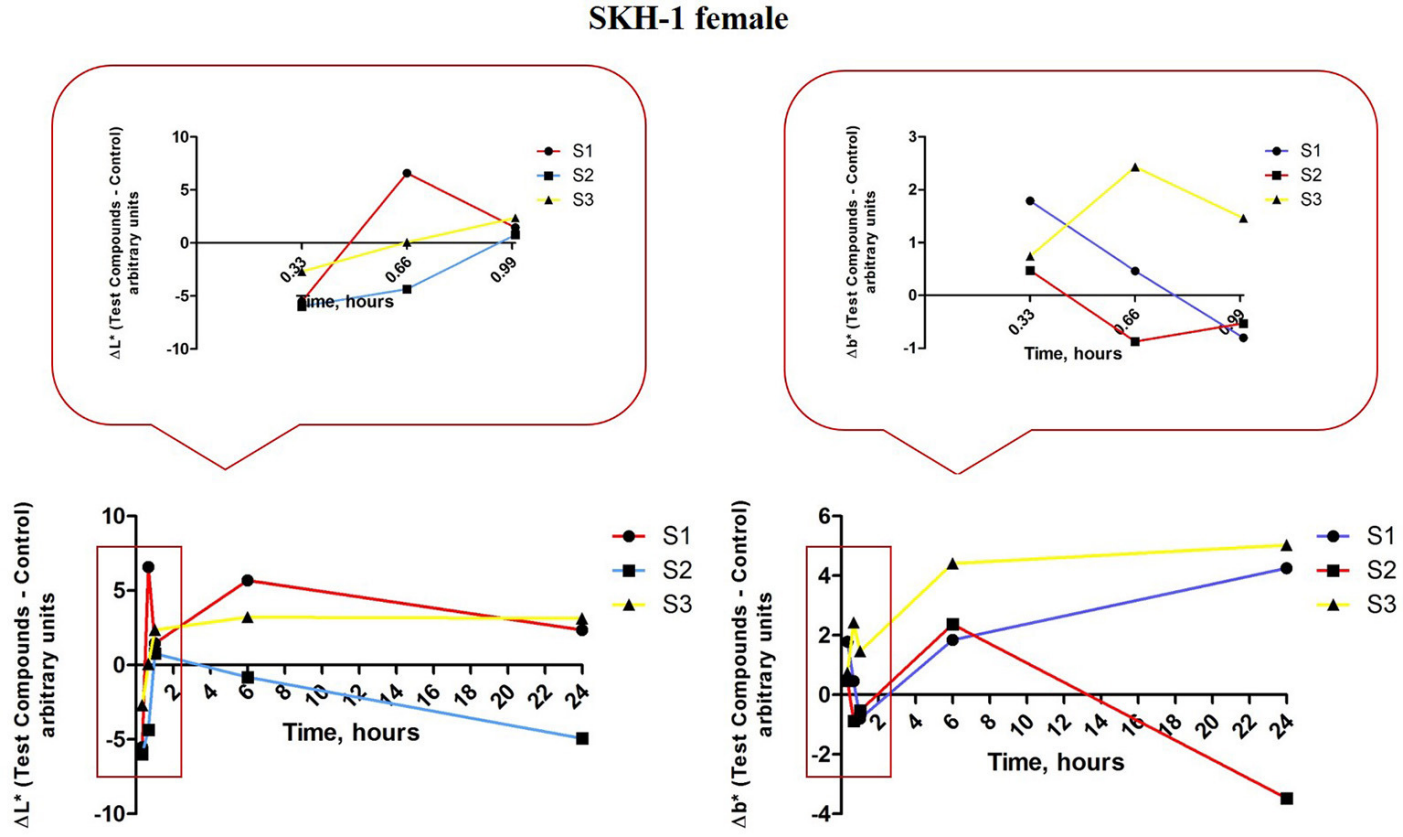
**Melanin-related measurements for SKH-1 females**. The values were expressed as ΔL^*^ and Δb^*^, Δ—is the difference between the mean value of the test group obtained for L^*^ and b^*^, respectively (for each time point) and the mean values for these coordinates in the control group (the values measured before the application of the suspensions).

For the females from group 1 that received S1 colloidal suspension in PBS (red line—Figure 11, left graph) there were observed the following aspects: ΔL^*^ value decreased after the first application of the S1 suspension (at 20 min), the second application led to an increase of this parameter (at 40 min) and the third application was accompanied by a decrease of ΔL^*^ value (at 60 min) as compared to the one recorded after the second application. At 6 h after the first application the ΔL^*^ value was similar with the one recorded after the second application (at 40 min), whereas at 24 h the value was in the same range with the one recorded after the third application (at 60 min).

In the case of Δb^*^ for the females from group 1 (S1 colloidal suspension topically applied—red line, Figure 11, right graph) the evolution of this parameter was as follows: the first application (at 20 min) led to an increased value as compared to the control (non—treated), a decrease after the second application (at 40 min) and the third application (60 min), an increase at 6 h and a reduced value at 24 h. The L^*^ (lightness or brightness) and b^*^–yellowness were described to be inversely correlated, an increase of L^*^ value was associated with the decrease of b^*^ value (and reverse) and the presence of pigmentation/augmentation of melanin content in the skin. Based on this correlation, our results indicated a pigmentation after the first application of S1 colloidal solution (low ΔL^*^ and high Δb^*^), after the second and third applications (high ΔL^*^ and low Δb^*^) the skin recovered and no pigmentation was recorded until the end of experiment.

Group 2 of female mice topically exposed to S2 colloidal solution—in distilled water presented some variations in the ΔL^*^ values during the experiment, especially in the first 24 h (blue line, Figure 11, left graph): a decrease after the first (at 20 min) and second (at 40 min) applications (more significant after the first application), an increase after the third application (at 60 min) followed by a reduced value at 6 and 24 h. An inversely evolution was recorded for Δb^*^ (blue line, Figure 11, right graph) values in the same group: an increase of the value after the first application (at 20 min), a decrease after the second (at 40 min) and third (at 60 min) applications, followed by increased values at 6 and 24 h. These data showed that application of S2 colloidal suspension induced a significant pigmentation/ an augmentation of melanin content after the first application, the skin tried to recover after the third application (high L^*^ and low b^*^), but the pigmentation became more intense after 6 and 24 h, respectively.

The results recorded for group 3 of female mice topically exposed to S3 colloidal suspension (maghemite) in distilled water concerning ΔL^*^ parameter (yellow line, Figure 11, left graph) showed: a decrease after the first application (at 20 min), followed by an increase after the second and the third applications, increase that continued in the same range at 6 and 24 h, too. In the case of Δb^*^ values, for group 3 of females mice were recorded only positive values, these values presenting a linear correlation with ΔL^*^ values (yellow line, Figure 11, right graph), data that were in contrast with the ones measured for the other two suspensions, S1 and S2, an explanation being the fact that S3 suspensions did not induce any changes in the melanin content.

**Figure 12:**
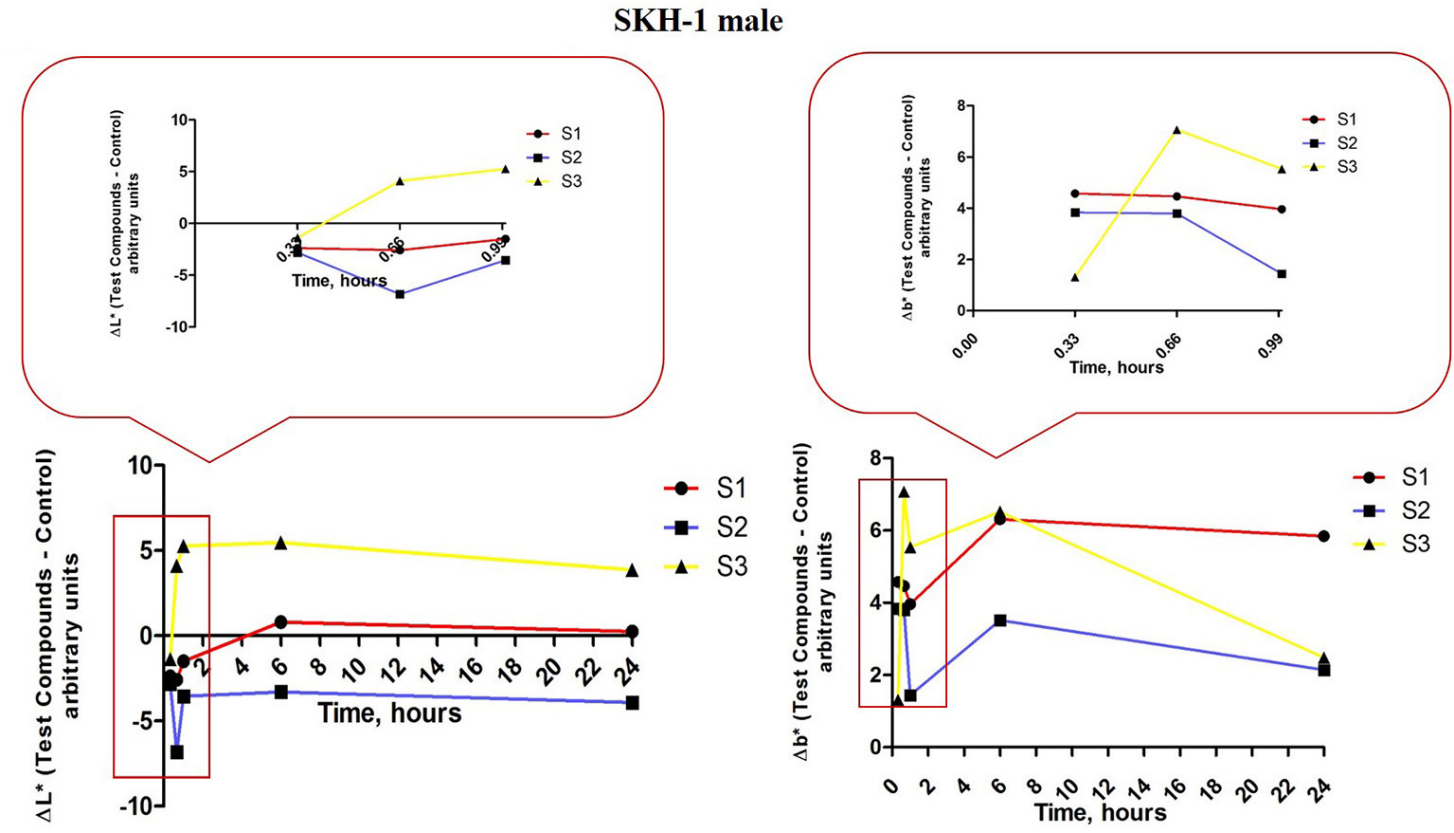
**Melanin-related measurements for SKH-1 males**. The values were expressed as ΔL^*^ and Δb^*^, Δ—is the difference between the mean value of the test group obtained for L^*^ and b^*^, respectively (for each time point) and the mean values for these coordinates in the control group (the values measured before the application of the suspensions).

In order to quantify the effect induced by the S1, S2 and S3 colloidal suspensions on melanin content in the male mice groups, the parameters L^*^ and b^*^ were measured. In the first group of male mice (topically treated with S1 colloidal suspension in PBS) was determined the following evolution for ΔL^*^ values (red line, Figure 12, left graph): all the three applications induced a decrease of ΔL^*^ (similar negative values), at 4 h after the first application the value of ΔL^*^ became positive reaching a maximum at 6 h and continued in the same range until 24 h. The values recorded for Δb^*^ (red line, Figure 12, right graph) in the same group of mice were in an inverse relationship with ΔL^*^ after the three applications of the S1 colloidal suspension (low L^*^ and high b^*^), while the values recorded at 6 and 24 h presented an ascending trend. These results could be explained as the development of a temporary pigmentation under the incidence of the applied suspensions followed by a recession of this status after 6 h post-application.

ΔL^*^ values measured in group 2 of male mice—exposed to S2 colloidal suspension in distilled water (blue line, Figure 12, left graph) exhibited the following tendency: a decrease after the 3 successive applications (negative values, the highest being recorded after the second application). The values at 6 and 24 h were closed to the ones calculated after the first and third applications. The other parameter evaluated Δb^*^ presented an evolution inversely correlated with the ΔL^*^ values (low L^*^ and high b^*^ and reverse; blue line, figure 12, right graph). These results showed that S2 suspension topically applied led to an elevation of melanin content in the skin of male mice, pigmentation that was conserved during the 24 h.

The results obtained in the case of group 3 of male mice exposed to S3 colloidal suspension in distilled water (maghemite; yellow line, Figure 12, left and right graphs) were similar with the ones obtained for group 3 of female mice showing a directly correlation between ΔL^*^ and Δb^*^, data that could be explained as no impact of this suspension—S3 on melanin content.

Another skin parameter measured in order to characterize the changes in the skin color post-application of the test suspensions was Δa^*^—redness or the green-red chromaticity coordinate related to erythema occurrence. Δa^*^ and ΔL^*^ are also interrelated in a linear inversely relationship, the lower the L^*^ value is, the higher the a^*^ value should be and also the degree of redness and erythema.

**Figure 13:**
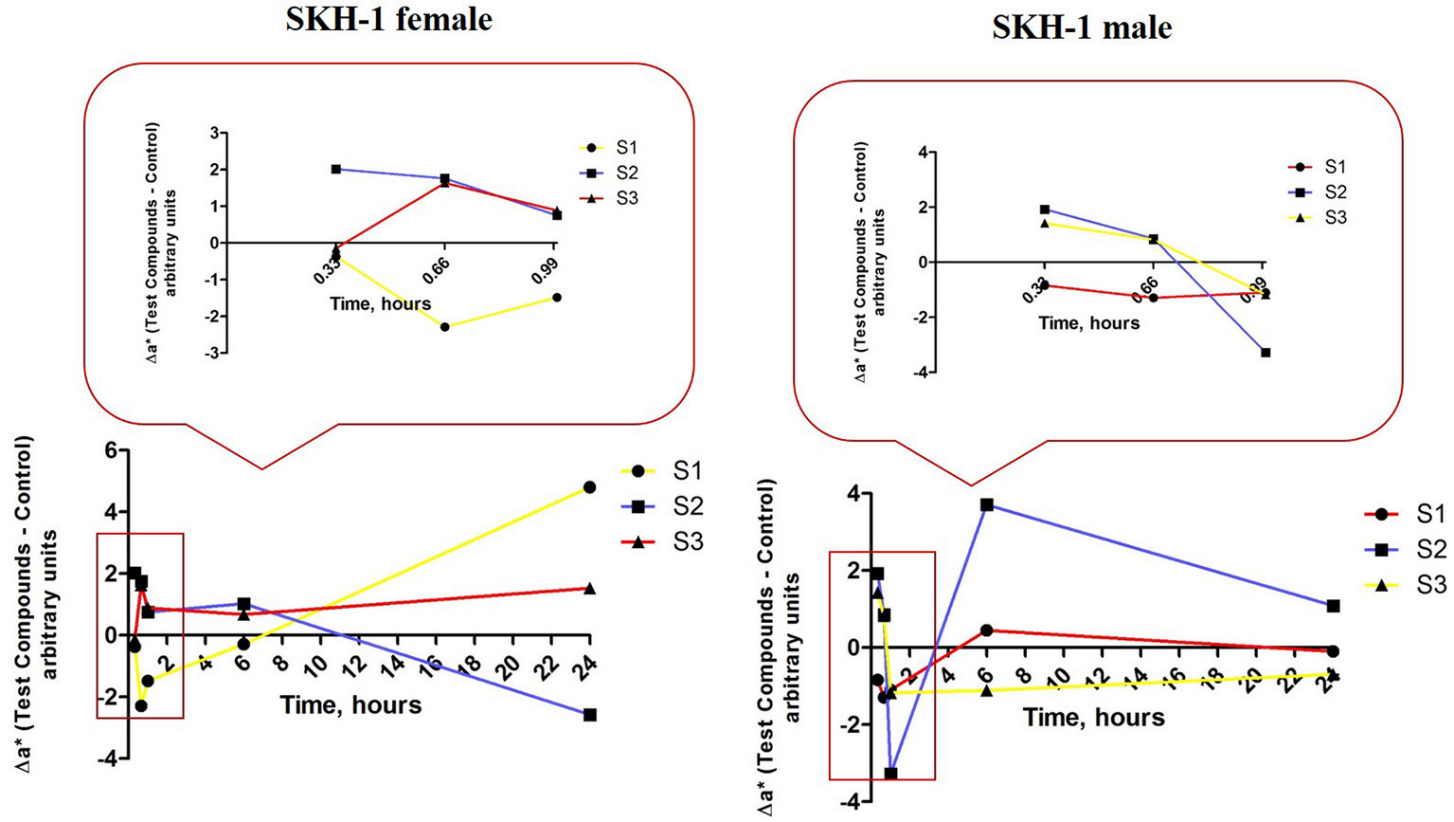
**Erythema-related measurements**. The values were expressed as Δa^*^, where Δ—is the difference between the mean value of the test group obtained for a^*^ (for each time point) and the mean value for this coordinate in the control group (the values measured before the application of the suspensions).

For group 1 of female mice (exposed to S1 suspension) the values of Δa^*^ that respected the inverse correlation with ΔL^*^ were only the values recorded after the second and the third applications (at 40 and 60 min), the others being in a direct correlation. The data could indicate that the erythema occurred starting with the second application and in time after 6 and 24 h the reaction faded and was retracted (red line, Figure 13, left graph).

The values calculated for the second group of female mice (exposed to S2 suspension) presented a similar tendency as the one observed to group 1, but the first signs of erythema were detected after the first application and the skin recovered after 6 h (blue line, Figure 13, left graph).

In group 3 of female mice (exposed to S3 suspension), the relationship between Δa^*^ and ΔL^*^ was a linear directly one, what could be associated with a lack of effect on this parameter (yellow line, Figure 13, left graph).

There were also observed some variations of the values calculated for Δa^*^ in the groups of male mice as compared to control values that were measured before the application of the suspensions.

The first group of mice (exposed to S1 suspension) presented a direct correlation between Δa^*^ and ΔL^*^ (high a^*^ and high L^*^) what could be explained as the absence of an impact on erythema values (red line, Figure 13, right graph). In the second group of male mice (exposed to S2 suspension), there were detected some signs of erythema after the first and the third applications (high a^*^ and low L^*^), but the erythema attenuated in time (at 6 h; blue line, Figure 13, right graph).

The male mice from group 3 (exposed to S3 suspension) presented signs of erythema starting with the first application and the signs were still visible after 24 h. These data could be explained by the inverse correlation between Δa^*^ and ΔL^*^ values calculated in this group (yellow line, Figure 13, right graph).

## Discussions

Iron oxide nanoparticles were found in different polymorphic phases, as: α, β, γ, δ, and ε—Fe_2_O_3_, maghemite (γ—Fe_2_O_3_) and magnetite (Fe_3_O_4_) being the two compounds mostly employed in biomedical applications. Magnetite and maghemite represent the first choice as magnetic nanoparticles for biomedical uses due to their unique features, such as: superparamagnetic properties *per se*–exhibit magnetism only in the presence of an external magnetic field, a single magnetic domain with a large constant magnetic moment and low toxicity (Muthukumaran and Philip, 2016; Velusamy et al., 2016). Magnetite was described to exceed the maghemite from the point of view of magnetic susceptibility and saturation magnetization (Muthukumaran and Philip, 2016).

Magnetite (Fe_3_O_4_) presents a cubic crystal structure of inverse spinel that has the Fe^2+^ cations located in the octahedral site (site B—surrounded by 6 oxygen anions) and the Fe^3+^ cations distributed evenly between the tetrahedral (site A—surrounded by 4 oxygen anions) and the B site (Li et al., 2010). This distribution of the cations is responsible for the total magnetization of the molecule (Marinca et al., 2016). Besides the metallic nanoparticles used as valuable tools to enhance the effectiveness of the current therapies and to increase the compliance of the patient to the treatments, there were described other types of nanoparticles engineered in this direction, such as: polymeric colloids, liposomes, solid lipid nanoparticles, cyclodextrins, and others (Kuskov et al., 2010; Medeiros et al., 2015; Soica et al., 2016).

The method used to synthesize iron oxide nanoparticles is considered a key factor for the future applications of the nanoparticles since the electrical, optical and magnetic features of these items are correlated to their size, parameter that is assigned during this process (Li et al., 2010). Other parameters that should be strictly controlled in the synthesis of iron oxide nanoparticles are shape, uniformity, crystallinity, and crystal structure (Jiang et al., 2014).

There were described some inconveniences concerning the methods used for the synthesis of iron oxide nanoparticles (poor monodispersity, irregular shape of the particles; Bloemen et al., 2012). Finding the proper method to obtain magnetic iron oxide nanoparticles with a reduced number of inconveniences related to the physico-chemical properties of the nanoparticles, in terms of stability, biocompatibility, the proper size and shape for biomedical uses, represented a real challenge for the researchers. A method that complies with most of these requirements was proved to be the solution combustion method. Solution combustion synthesis was described as a simple, universal and low time consuming method that could be applied for the preparation of a variety of nanosize materials. This type of procedure it's based on a self-sustained reaction that requires the presence of oxidants (homogenous solutions) and fuels (like: urea, glycine, hydrazides, etc.). The resultant products of this procedure are nanosize oxide materials, but this method also delivers a homogeneous concentration of trace amounts of rare-earth impurity ions in a single step. Solution combustion synthesis proved to be an efficient method for the procurement of different metal nanopowders, such as: Ni, Zn, Cd, Al, Ti, Cu, Fe, etc. (reviewed in Aruna and Mukasyan, 2008; Ianoş et al., 2012, 2014; Huang et al., 2016).

In the present study, it was applied the solution combustion synthesis in order to obtain the magnetic iron oxide nanoparticles. The major advantage of the combustion method is that the final reaction product is obtained directly after combustion, without subsequent calcinations, therefore the energy consumption is reduced. Phase composition, morphology, and reactivity of the synthesized powders are established based on the application of specific requirements and controlled by synthesis conditions. In a study developed by Gupta and Wells it was shown the selective behavior of the magnetic iron oxide nanoparticles obtained by the means of combustion synthesis (toxic for tumor, but protective with normal cells), these results offering a new perspective on the potential use of these magnetic nanoparticles in cancer therapy (Gupta and Wells, 2004).

Ianos and co-workers proposed an adapted protocol for the solution combustion synthesis in order to obtain nano-scaled iron oxide nanoparticles (magnetite and maghemite; Ianoş et al., 2012, 2014). For the synthesis of the iron oxide nanoparticles analyzed in this study it was applied the protocol described by Ianos et al. By employing the same method, the results that were obtained in terms of physico-chemical properties are similar with the ones showed by Ianoş et al. (2012; 2014), as follows: for magnetite (D_XRD_—crystallite size = 18; S_BET_—specific surface area = 56; D_BET_—particle diameter from BET = 21; M_*s*_—saturation magnetization = 57.7; M_r_—remanent magnetization = 4.5; H_c_—coercivity = 5.2) and for maghemite (D_XRD_—crystallite size = 5; S_BET_—specific surface area = 149; D_BET_—particle diameter from BET = 8; M_s_—saturation magnetization = 41.5; M_r_—remanent magnetization = 0.7; H_c_—coercivity = 1). Considering the magnetic properties of the resulted powders, it was observed that the saturation magnetization of the samples S1, S2 prepared by combustion synthesis was slightly higher than that of the sample S3 which after combustion was washed with oxygenated water in order to remove the carbon from the magnetic nanoparticles surface. At the same time, the remanent magnetization and the coercivity of combustion synthesized magnetic nanoparticles were very close to the superparamagnetic behavior. The specific surface area of sample S3 was the highest, which mean that the particles are very small, fact that was also observed from the BET diameter. This aspect indicated that these particles, obtained by combustion method, having narrow size distribution with a size range of <50 nm, can be successfully used for intravenous administration (i.v.), not only as magnetic resonance imaging (MRI) contrast agents, vectors for gene and drug delivery, or as agents for hyperthermia therapy (Boyer et al., 2010; Estelrich et al., 2015).

One of the problems encountered in the synthesis of iron oxide nanoparticles is the aggregation process characteristic for the nanoscale particles with a large surface-to-volume ratio. The solution proposed for this matter was the use of stabilizing agents or coating agents that adhere to the surface of the nanoparticles and offer a spatial isolation leading to the achievement of monodisperse nanoparticles (Li et al., 2010). To avoid such agglomeration and coagulation of the nanoparticles, they are coated with specific surfactants that present in their structure a hydrophobic element and a polar group. The hydrophobic element is adsorbed on the surface of the nanoparticles whereas the polar group enwidens into the water solution and protects the nanoparticle against agglomeration, process that is known as functionalization of the nanoparticle (Medeiros et al., 2015).

To obtain biocompatible colloidal suspensions of iron oxide nanoparticles, in this study it was proposed as coating agent a fatty acid—the oleic acid. Oleic acid (OA) is frequently used as a capping agent for the iron oxides nanoparticles, because it can form a dense protective monolayer, which is strongly bonded to the surface of nanoparticles, thus being produced monodisperse and uniform nanoparticles. If the iron oxide nanoparticles are monolayer—coated with OA, these will be dispersible only in organic solvents thereby limiting their use for biomedical applications (Patil et al., 2014). A single layer of OA is adequate for loading different hydrophobic drugs, but is not biocompatible and appropriate for medical use due to the presence of hydrophobic surfaces with a large surface area that might lead to considerable particles size (aggregation and big clusters formation) and recognition by the immune cells and clearance. In order to obtain biocompatible iron oxide nanoparticle suspensions is required the addition of a hydrophilic surfmer to the oleic acid monolayer used for functionalization of the nanoparticles (Tran et al., 2015).

The novelty of the method that was proposed to prepare biocompatible colloidal suspensions of iron oxide nanoparticles in the present study consisted in coating with double layer of OA the iron oxide nanoparticles resulted during the solution combustion synthesis, method that was not described in the literature, not to our knowledge. The coating process was conducted in compliance with the protocol described by Bica et al. (2007) with several modifications and there were obtained OA double coated iron oxide nanoparticles that were characterized in terms of DLS and TEM analyses.

The values that were measured for the particles size dimensions of the three samples analyzed (S1—63 nm, S2—43 nm, S3—80 nm—Figure 1) by the means of DLS indicate that the coated nanoparticles have a very narrow size distribution range with superparamagnetic behavior at room temperature. The hydrodynamic diameter of the coated nanoparticles is higher as compared to the values measured for the pure/uncoated nanoparticles, and this could be a confirmation of the coating process. Furthermore, the nanoparticles are stable in PBS/distilled water at neutral pH. Based on these data, it could be said that the colloidal suspensions of the OA double coated iron oxide nanoparticles obtained are suitable for *in vitro* and in *vivo* applications, their dimensions being in the range established for biomedical domain, an average particle's diameter smaller than 100 nm and the polydispersity 0.1 (Shete et al., 2015; Medeiros et al., 2015).

According to the TEM measurements and micrographs (Figure 3) concerning the morphology and ultrastructure of the colloidal suspensions, the results confirmed that the prepared iron oxide particles were of nanosize, similar results being described by Buzea et al. (2007). The values recorded for the size of the nanoparticles from all the samples (see Figure 4) are in the range of 7–22 nm, what could indicate the presence of high polydispersity, results that are in accordance with the data obtained from DLS measurements. It is known that DLS measures the size of the aggregates not of a single iron oxide particle and this could be an explanation for the differences between the values of average particle diameter measured by DLS technique and the ones recorded by TEM. Our results are in agreement with the data from the literature (Li et al., 2010; Medeiros et al., 2015; Shete et al., 2015).

A considerable number of studies used oleic acid in monolayer as coating agent for different nanoparticles engineered for targeted drug delivery and release and the results showed that this fatty acid played a crucial role in controlling the shape, monodispersity and thermal stability of the nanoparticles (Li et al., 2010; Tran et al., 2015; Muthukumaran and Philip, 2016; Velusamy et al., 2016). Jiang et al. proved that the shape and size of the magnetic iron nanoparticles synthetized by high-temperature decomposition was influenced by the concentration of oleic acid used as coating agent: the higher the concentration of OA, the wider the particle size and of irregular shapes (Jiang et al., 2014). Similar results regarding the impact of oleic acid on particle size and shape dependent on concentration of acid used, were described by Soares et al. in a study published in 2015 (Soares et al., 2015). The group of Marinca proposed a novel method for the synthesis of magnetite nanoparticle coated with oleic acid for biomedical purposes, which proved to be a combination between the ceramic method and wet mechanical milling and the results obtained highlighted the dual role of oleic acid as surfactant and to prevent the coalescence of the nanoparticles. Moreover, OA coating limits the contamination of the resulted powder with iron and forbids the reaction between magnetite and iron (Marinca et al., 2016).

The bond between the first layer of oleic acid and the iron oxide nanoparticles could be explained by the interaction between the carboxyl group from the oleic acid structure and the surface of the iron oxide, probably through a coordination of the iron atoms and both oxygen from the carboxyl group. The hydrocarbon tail of the oleic acid remains free to interact with the second surfactant—the surfmer (the active surfactant) and leads to a solubilization of the coated iron oxide nanoparticles in organic solvents (Medeiros et al., 2015; Shete et al., 2015). By adding another layer of oleic acid to the iron oxide nanoparticles coated with a first layer of oleic acid, it was achieved an aqueous suspension of iron oxide nanoparticles with a long stability (more than 3 months; Lan et al., 2007; Li et al., 2010).

The group of Ingram obtained magnetite nanoparticles by co-precipitation method, that were further coated with a double layer of oleic acid what provided them colloidal stability in water at pH = 7–10 and proved to be efficient to stabilize the oil-water emulsions with applicability in magnetic imaging and sensing applications (Ingram et al., 2010). Our results are in concordance with the data existent in the literature, since we obtained stable water dispersible suspensions of OA double coated iron oxide nanoparticles.

Concerning the antioxidant activity assessment, it was observed that for an amount of 52 mg of Fe_3_O_4_ (sample S2) the AOA obtained was 20% for the dilution 1:100. This value is similar to the one obtained in the case of using 72 mg γ-Fe_2_O_3_. Paul et al. investigated the antioxidant properties of iron oxide particles of different sizes and showed that the free radical scavenging efficiency of bare iron oxide nanoparticles (α-Fe_2_O_3_) was found to be almost 50% for DPPH by 200 mg of iron oxide nanoparticles (Paul et al., 2009). Bhattacharya et al. found a very high free radical scavenging activity of 89% by using 10 mg of α-Fe_2_O_3_/C nanocomposites. This can be explained by the electron transfer from the nanocomposite system toward the free radical located at the nitrogen atom in DPPH (Bhattacharya et al., 2014).

A matter of great concern for the use of nanoparticles in biomedical field is considered the incomplete toxicological profile of these nanomaterials, the toxicity data being somehow controversial. A considerable number of studies were conducted in order to verify the toxicity induced by the nanoparticles, both *in vitro* and *in vivo*, but also in the other domains that use nanotechnology/nanoparticles (agriculture, food sector; Balmuri et al., 2016; Bostan et al., 2016; Neagu et al., 2016; Piperigkou et al., 2016; Valdiglesias et al., 2016).

One of the objectives suggested in this study consisted in the *in vitro* and *in vivo* toxicological evaluation of the biocompatible colloidal suspensions obtained in order to establish their safety profile.

Taking into consideration the data that were obtained in the section of physico-chemical characterization of the novel OA double-layered iron oxide nanoparticles colloidal suspensions, it was tested the effect of these suspensions *in vitro* on human keratinocytes (HaCat cell line) viability. The test was performed using Alamar blue assay. This assay relies on the capacity of the metabolically active cells (living cells) to reduce resazurin, the active compound from Alamar blue solution in order to quantitatively measure the number of viable normal or cancer cells after stimulation with test compounds (Riss et al., 2016). Our results presented in Table 3 indicated, in the case of all three colloidal suspensions (magnetite and maghemite oleic acid double coated nanoparticles), a lack of toxicity induced by the iron oxide nanoparticles colloidal suspensions after an exposure of 24 h at the concentrations used (5, 10, and 25 μg·mL^−1^).

Similar results were obtained by Naseroleslami et al. when tested different concentrations (in the range of 25–800 μg·mL^−1^) of PEGylated superparamagnetic iron oxide nanoparticles obtained by co-precipitation method on human-derived amniotic membrane stem cells, the cell viability at the lowest concentration (25 μg·mL^−1^) being 99.96 ± 0.05% independent of exposure time (24, 48, and 72 h; Naseroleslami et al., 2016). In a recent study developed by Joris and co-workers, concerning the safety profile of iron oxide nanoparticles in neural cells of different origin (human and mouse) and type (stem cells, progenitor cell line and cancer cell line), it was shown that iron oxide nanoparticles coated with an amphiphilic polymer—poly(isobutylene-alt-maleic anhydride)-graft-dodecyl (PMA) induced the lowest loss of cell viability as compared to gold and silver nanoparticles. The highest susceptibility to acute toxicity was observed in the case of human neural stem cells followed by the mouse stem cells, whereas the least susceptibility was recorded for cancer cells (Joris et al., 2016). Shete et al. tested both uncoated and coated magnetite nanoparticles on L929 cell line (mouse fibroblasts) and it was observed a very low cytotoxicity even after a period of 48 h stimulation with different concentrations (0.1, 0.5, 1.0, 1.5, and 2.0 mg·mL^−1^; Shete et al., 2015).

Pongrac et al. demonstrated that poly(L-lysine)-coated maghemite nanoparticles after a stimulation of 48 h led to a cell viability and proliferation around 80% at a concentration of 0.2 mg·mL^−1^ while a concentration similar (0.03 mg·mL^−1^) with the highest concentration used in the present study (25 μg·mL^−1^) was responsible for around 5% dead neural stem cells—NSC (Pongrac et al., 2016), results that indicated a reduced degree of toxicity induced by the iron nanoparticles.

There were also published data that affirmed the toxicity induced by the iron oxide nanoparticles, the noxious effects being attributed to the difference in particles size and shape, the dose used for stimulation, and the cell type (reviewed in Valdiglesias et al., 2016).

Another parameter tested in order to verify the *in vitro* effects of the colloidal suspensions of magnetite and maghemite was their impact on cells migration and proliferation. According to our results (Figures 7, 8, 9), the suspensions had no effect on HaCat cells migration at all the concentrations tested, nor after 3 h, neither after 24 h stimulation. Our data are in agreement with the data presented by Muhammad et al. who showed that stimulation with SPIO (superparamagnetic iron oxide) nanoparticles of adipose-derived mouse stem cells and bone marrow-derived mouse stem cells did not impaired cell migration (Muhammad et al., 2015).

The *in vitro* results that were obtained could be considered a toxicological profile for the coating agent, in this case oleic acid, since the iron oxide nanoparticles were not degraded in the timeframe of the cytotoxicity assay—24 h.

Since the colloidal suspensions that were obtained in the present study, represent an element of originality (no biological studies were performed to the best of our knowledge on iron oxide nanoparticles obtained by solution combustion synthesis and coated with a double layer of oleic acid), to test their effects *in vivo* became mandatorily.

In this study it was conducted a test of dermal acute toxicity of the colloidal suspensions on female and male SKH-1 hairless mice, toxicity that was verified after topical applications of the suspensions. It was decided to perform this kind of toxicity test based on the fact that the preliminary data obtained will represent the fundamental basis for further *in vivo* studies regarding the formulation of topical forms using iron oxide nanoparticles as carriers for agents effective in different skin pathologies. Our data showed that topical applications of S1, S2, and S3 suspensions had no effect on mice body weight or on behavioral patterns.

In order to evaluate the effects induced by these colloidal suspensions at skin level, there were measured and analyzed several physiological skin parameters, including: skin hydration, melanin content and erythema. The method applied was a non-invasive assay, using the equipment from Courage-Khazaka Electronics GmbH: Corneometer®CM 825 and Skin Colorimeter CL 400.

Topical application of S1 colloidal suspension in PBS led to a decrease of skin hydration in the females group, but the initial values of the parameter were achieved during the first 24 h post-application (red line, Figure 10, left graph). These modifications were not detected in the male mice group that suffered the same protocol (red line, Figure 10, right graph). The S2 and S3 aqueous suspensions induced an increase of skin hydration in both female and male mice groups, hydration that was maintained during the first 24 h (blue and yellow lines, Figure 8).

The differences between female and male mice concerning the skin hydration recorded in the present experiment can be explained by the sex disparities in skin structure: the males present a lower pH value, a higher sebum content and skin hydration values, and also a higher susceptibility to develop skin pathologies, including cancer (Boelsma et al., 2003; Dehelean et al., 2016).

The tristimulus colorimetry method was applied in order to verify the changes of skin color associated to the topical application of test suspensions. This type of method is commonly used in dermato-cosmetic research, the parameters investigated, melanin content and erythema being indicators of skin barrier integrity and sensitivity after contact with different substances, drugs or vehicles (Matias et al., 2015). This technique offers quantitative measurements of skin color expressed with the help of a 3-digit output L^*^, a^*^ and b^*^—arbitrary values, system that is recognized by the International Commission of Illumination (Commission Internationale de L'Eclairage—CIE; Alaluf et al., 2002; Tzung et al., 2009). L^*^ is known as lightness, brightness, or level of darkness and can take values in the interval 0 and 100, the value 0—is black and 100 is white and is associated with the melanin content from the external skin layers. a^*^ can reach negative or positive values between −60 and + 60, from green to red, this marker being known as redness and is considered an indicator for the presence of the erythema (the higher the values is, the erythema is more intense). b^*^—yellowness, the coordinate that is related to melanin content from the skin, can be expressed as negative or positive values from −60 (blue) to +60 (yellow; Alaluf et al., 2002; Tzung et al., 2009; Yang et al., 2016).

There was described an inverse correlation between the L^*^ and a^*^ and b^*^ values. The darkest type of skin presents a low L^*^ value and high values for a^*^ and b^*^ (Alaluf et al., 2002). Our results indicated that in the group 1 of female mice was observed a pigmentation after the first application of S1, whereas the erythema signs were detected only after the second and third application. Both pigmentation and erythema faded until the end of 24 h. These data showed that application of S1 suspension on female mice skin led to some changes of skin parameter, but the change was not significant in terms of disturbing the integrity of skin barrier function.

In the case of the second group of female mice—exposed to S2 suspension it was detected a pigmentation that lasted for 24 h while the erythema signs were recorded after the first application, but after 6 h the erythema values were decreased. These dates could be explained by the fact that S2 suspension was better absorbed in the skin being an aqueous solution, what led to a pigmentation for 24 h. The S3 suspension had no impact in the group of female mice neither on the parameter related to melanin content (L^*^ and b^*^), nor on the a^*^—the coordinate related to erythema.

The results recorded in the groups of male mice were as follows: S1—application was associated with a temporary pigmentation after the applications of the suspension (<6 h), but no sign of erythema was recorded; S2—application led to an increase of melanin content value that was maintained constant in the 24 h and it was observed a slight erythema after the application (only for 6 h) and S3—application had no impact on melanin content, but there were recorded erythema signs after the topical applications.

The changes observed in the skin parameters values showed a slight disturbance associated to the topical applications of the iron oxide nanoparticles, but these changes were temporary and should not be interpreted as signs of toxicity.

On account of our group expertise in the field of skin pathologies (Dehelean et al., 2013, 2016; Gheorgheosu et al., 2013; Soica et al., 2014; Danciu et al., 2015), the present study could be seen as a first step in the synthesis and characterization of the magnetic iron nanoparticles double coated with oleic acid as future carrier platforms for transdermal drug delivery in skin malignancies (melanoma and non-melanoma skin cancers, auto-immune diseases—epidermolysis bullosa acquisita). In this regard was evaluated the toxicological profile of the colloidal suspensions on human keratinocytes cell line and by applying the dermal acute test to SKH-1 hairless mice.

The transdermal drug delivery offers several advantages as compared to the other routes of administration (oral and intravenous): a controlled release of the drug, avoidance of the first hepatic metabolism and a higher patient compliance by reducing the pain associated to i.v. administration. Oleic acid is a FDA recognized agent for increasing skin permeation and it is frequently applied in different commercial formulations. The mechanism of action of oleic acid consists in: interaction with the lipid content of the *stratum corneum* what leads to the changes in the lipid bilayer, characterized by the apparition of some pools responsible for the defects that appear in the permeability and it is facilitated the entry of different molecules into the profound skin layers (Shah et al., 2012).

All these data support the idea of oleic acid double coated iron oxide nanoparticles as promising carrier platforms for drug delivery in skin disorders.

## Conclusions

In the current study were obtained biocompatible colloidal suspensions based on iron oxide nanoparticles prepared by the means of solution combustion synthesis and coated with a double layer of oleic acid. TEM and DLS analyses confirmed their nanosize features (an average particle diameter around 7–22 nm) what makes them suitable for biomedical applications, in addition to their high stability and solubility in aqueous solutions. Magnetite solubilized in distilled water (sample 2) exhibited an antioxidant activity at the concentrations tested. The *in vitro* evaluations of the colloidal suspensions tested indicated a lack of toxicity on human keratinocytes cell viability, proliferation, and migration. The *in vivo* acute dermal toxicity test revealed some changes in the values of physiological skin parameters, but not significant as to interfere with the skin barrier function. These data offer valuable information for future studies regarding the use of iron oxide nanoparticles as carrier platforms for drug delivery in skin pathology.

## Author contributions

EM, IP, and CP—effectuated the synthesis of the iron oxide nanoparticles and preparation of the colloidal suspensions, the physico-chemical characterization, analysis of the data and drafting the work. DC and CD—contribution at the conception of the study, performed the *in vitro* and the *in vivo* tests, analysis and interpretation of the data acquired, drafting the work and prepared the manuscript for submission. CM—performed TEM assay, acquired and analyzed the data, drafting the work. CS, CC, VT, and AT—elaboration of the final version of the manuscript, correction of the language, analysis of the data and revised critically the work.

## Funding

This work was supported by a grant of the Romanian National Authority for Scientific Research and Innovation, *CNCS – UEFISCDI*, project number PN-II-RU-TE-2014-4-2842.

### Conflict of interest statement

The authors declare that the research was conducted in the absence of any commercial or financial relationships that could be construed as a potential conflict of interest.

## Abbreviations

AOA: antioxidant activity
CIE: Commission Internationale de L'Eclairage
DBET: particle diameter from BET
DLS: dynamic light scattering
DMEM: Dulbecco's modified Eagle Medium
DPPH: 1,1-Diphenyl-2-picryl-hydrazyl
DXRD: crystallite size
FBS: Foetal Bovine Serum
HaCat cells: human keratinocytes cell line
Hc: coercivity
Mr: remanent magnetization
MRI: magnetic resonance imaging
Ms: saturation magnetization
OA: oleic acid
PBS: phosphate saline buffer
SBET: specific surface area
TEM: transmission electron microscopy
i.v.: intravenously administration.
